# Ischaemic post‐conditioning in rats: Responder and non‐responder differ in transcriptome of mitochondrial proteins

**DOI:** 10.1111/jcmm.15209

**Published:** 2020-04-16

**Authors:** Rolf Schreckenberg, Johann Klein, Hanna Sarah Kutsche, Rainer Schulz, Kamilla Gömöri, Péter Bencsik, Bettina Benczik, Bence Ágg, Éva Sághy, Péter Ferdinandy, Klaus‐Dieter Schlüter

**Affiliations:** ^1^ Department of Physiology Justus Liebig‐University Gießen Germany; ^2^ Department of Pharmacology and Pharmacotherapy University of Szeged Szeged Hungary; ^3^ Pharmahungary Group Szeged Hungary; ^4^ Cardiometabolic and MTA-SE System Pharmacology Research Group Department of Pharmacology and Pharmacotherapy Semmelweis University Budapest Hungary

**Keywords:** miRNA, post infarct remodeling, UCP, VEGF

## Abstract

Ischaemic post‐conditioning (IPoC) is a clinical applicable procedure to reduce reperfusion injury. Non‐responsiveness to IPoC possibly caused by co‐morbidities limits its clinical attractiveness. We analysed differences in the expression of mitochondrial proteins between IPoC responder (IPoC‐R) and non‐responder (IPoC‐NR). Eighty rats were randomly grouped to sham, ischaemia/reperfusion (I/R), IPoC or ischaemic pre‐conditioning (IPC, as positive cardioprotective intervention) in vivo. Infarct sizes were quantified by plasma troponin I levels 60 minutes after reperfusion. After 7 days, rats were sacrificed and left ventricular tissue was taken for post hoc analysis. The transcriptome was analysed by qRT‐PCR and small RNA sequencing. Key findings were verified by immunoblots. I/R increased plasma troponin I levels compared to Sham. IPC reduced troponin I compared to I/R, whereas IPoC produced either excellent protection (IPoC‐R) or no protection (IPoC‐NR). Twenty‐one miRs were up‐regulated by I/R and modified by IPoC. qRT‐PCR analysis revealed that IPoC‐R differed from other groups by reduced expression of arginase‐2 and bax, whereas the mitochondrial uncoupling protein (UCP)‐2 was induced in IPC and IPoC‐R. IPoC‐R and IPoC‐NR synergistically increased the expression of non‐mitochondrial proteins like VEGF and SERCA2a independent of the infarct size. Cardiac function was more closely linked to differences in mitochondrial proteins than on regulation of calcium‐handling proteins. In conclusion, healthy rats could not always be protected by IPoC. IPoC‐NR displayed an incomplete responsiveness which is reflected by different changes in the mitochondrial transcriptome compared to IPoC‐R. This study underlines the importance of mitochondrial proteins for successful long‐term outcome.

## 1 INTRODUCTION

Today, early reperfusion is the key strategy to overcome acute ischaemia/reperfusion (I/R) injury. However, although successful reperfusion greatly improves the acute outcome, the long‐term prognosis of patients depends on subsequent cardiac remodelling.^1^ Procedures such as ischaemic pre‐conditioning (IPC) or ischaemic post‐conditioning (IPoC) have been shown to reduce reperfusion injury and should improve also the long‐term outcome. In clinical routine, IPoC is more relevant than IPC, but although studied for years rather extensively it is not yet established in clinical routine. One of the great disadvantages is the relative high number of non‐responders.^2^ Although this is often associated to co‐morbidities such as advanced age, dyslipidemia, diabetes or hypertension as well as to patient‐specific medications neither clinical nor pre‐clinical studies show a unique relationship between IPoC and protection.^3^, ^4^, ^5^ Specifically, in rats, different reports found either no or small protection^6^, ^7^, ^8^, ^9^ or effective protection^10^, ^11^, ^12^, ^13^, ^14^ with and without co‐morbidities.^15^, ^16^, ^17^, ^18^

In order to explain the large outcome differences in pre‐clinical studies, it may be argued that differences between protocols, source of animals, sex and age of animals, animal handling and other variables are responsible for inconsistent results. To overcome these limitations, we started a study in which experimental variables were minimized. Nevertheless, rats undergoing IPoC showed heterogeneous responsiveness separating them in true responders and non‐responders. Transcriptome analysis was used as an unbiased evaluation according to the ESC guidelines to identify differences between both groups.^19^ The analysis revealed that IPoC similarly affected transcriptome response in non‐mitochondrial proteins but displayed significant differences in the transcriptome of mitochondrial proteins. As a potential source of regulation, differences in mitochondrial transcriptome were ale found for microRNA (miR) related to mitochondria.

## 2 MATERIALS AND METHODS

The investigation confirms the Guide for the Care and Use of Laboratory Animals published by the US National Institute of Health (NIH Publication No. 85‐23, revised 1996). The study was approved by the local ethics committee of the University of Szeged.

### 2.1 Rat model of ischaemia and reperfusion

Female Wistar‐Hannover rats weighing 215‐265 g were anaesthetized by intraperitoneal injection of 60 mg/kg sodium pentobarbital (Euthasol, Produlab Pharma b.v.). Animals were intubated and mechanically ventilated (Model 683; Harvard Apparatus) with room air in a volume of 6.2 mL/kg and a frequency of 55 ± 5 breath/min according to bodyweight. Rats were placed in supine position on a heating pad to maintain body core temperature in physiological range (37.0°C ± 1.0°C). Body surface electrocardiogram (ECG) was monitored throughout the experiments (Haemosys, Experimetria Inc). Left anterior descending coronary artery (LAD) occlusion was induced by a left thoracotomy. A 5‐0 Prolene suture (Ethicon, Johnson & Johnson Kft. Hungary) was placed around LAD artery and a small plastic knob, which was threaded through the ligature and placed in contact with the heart, was used for making occlusion for 30 minutes. The presence of ischaemia was confirmed by the appearance of ST segment elevation and ventricular arrhythmias. After 30‐minute ischaemia, the occlusion was released and the suture was removed from the heart. Restoration of blood flow was confirmed by the appearance of arrhythmias observed in the first minutes after the onset of reperfusion. Then, the chest was closed in layers with a 4‐0 Monocryl suture (Ethicon, Johnson & Johnson Kft. Hungary) and 0.3 mg/kg nalbuphin (Nalbuphin Orpha, Torrex Chiesi Pharma GmbH) was given sc. to alleviate post‐operative pain. One hour after the onset of reperfusion, venous blood was sampled from the right femoral vein for troponin I determination. Rats were allowed to recover for 7 days after coronary occlusion.

### 2.2 Experimental groups

**Figure 1:**
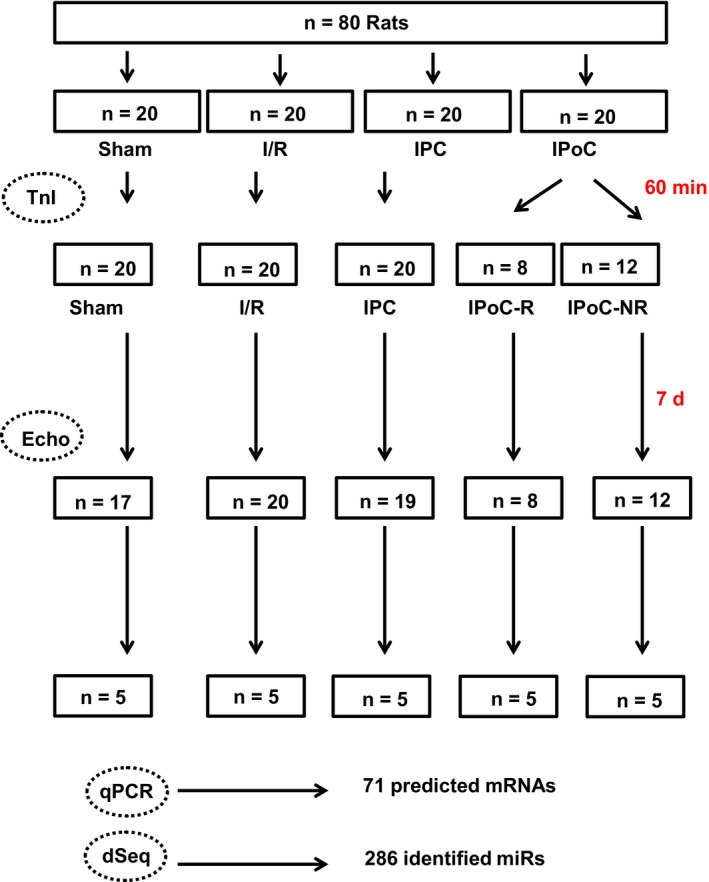
Study protocol. TnI = Quantification of plasma troponin I levels; Echo = functional analysis via echocardiography; qPCR, quantitative real‐time RT‐PCR analysis; dSeq = deep sequencing for analysis of miRs

Animals were randomly assigned to four experimental groups: (a) sham operated, (b) ischaemic control, (c) ischaemic pre‐conditioning (IPC) and (d) ischaemic post‐conditioning (IPoC). Sham operated rats underwent the same procedure detailed above without occluding the LAD. IPC was performed by occluding the LAD in three cycles for 3 minutes interrupted with 5 minutes perfusions prior to 30‐minute coronary occlusion. IPoC was performed by three cycles of 10‐second reperfusion/10‐second re‐occlusion at the end of 30‐minute coronary occlusion. Figure 1 gives an overview about the experimental design. The study protocol confirm to the recommendation given previously.^20^

### 2.3 Echocardiography

Cardiac morphology and function were also assessed by transthoracic echocardiography in the post‐ischaemic heart 7 days after 30‐minute occlusion and reperfusion of the LAD. Echocardiography was performed as described previously.^21^, ^22^ The observer was blinded to the animal groups. Briefly, rats were anaesthetized with sodium pentobarbital (Euthasol, 40 mg/kg bodyweight, i.p.), the chest was shaved and the animal was placed in supine position onto a heating pad. Two‐dimensional and M‐mode echocardiographic examinations were performed in accordance with the criteria of the American Society of Echocardiography with a Vivid 7 Dimension ultrasound system (General Electric Medical Systems) using a phased array 5.5‐12 MHz transducer (10S probe). Data of three consecutive heart cycles were analysed (EchoPac Dimension software, General Electric Medical Systems) and then the mean values of the three measurements were calculated and used for statistical evaluation. Systolic and diastolic wall thicknesses were obtained from parasternal short‐axis view at the level of the papillary muscles. The left ventricle diameters were measured by means of M‐mode echocardiography from short‐axis views between the endocardial borders. Functional parameters including fractional shortening and ejection fraction were calculated on short‐axis view images.

### 2.4 Measurement of cardiac troponin I release in plasma

Plasma samples were collected into heparinized tubes (Sarstedt) from the femoral vein at the 60th minute of reperfusion, and plasma was separated to determine cardiac troponin I (TnI) release after acute myocardial infarction. Plasma TnI concentration was determined by a conventional ELISA kit (Life Diagnostics, Inc) according to the recommendations of the manufacturer. Briefly, plasma samples were diluted 4 to 40 times according to the treatment protocol (ie, sham or ischaemic) and to previous pre‐analyses to get absorbances in the range of standard absorbances. Diluted samples were allowed to react simultaneously with 2 antibodies against rat TnI (1 is immobilized on the microtitre wells, and the other is conjugated to horseradish peroxidase [HRP] in soluble phase), resulting in TnI being sandwiched between the solid phase and HRP‐conjugated antibodies. After 1 hour of incubation at room temperature on a plate shaker, the wells were washed with wash solution to remove unbound HRP‐conjugated antibodies. A solution of tetramethylbenzidine, a HRP substrate, was then added and incubated for 20 minutes, resulting in the development of a blue colour. The colour development was stopped by addition of 1 N HCl, which changed the colour to yellow. The concentration of TnI was proportional to the absorbance at 450 nm.

### 2.5 RNA isolation and real‐time RT‐PCR

Total RNA was isolated from the LV using peqGold TriFast (peqlab, Biotechnologie GmbH) according to the manufacturer's protocol. To remove genomic DNA contamination, RNA samples were treated with 1 U DNase/µg RNA (Invitrogen) for 15 minutes at 37°C. One microgram of total RNA was used in a 10 µL reaction to synthesize cDNA using Superscript RNaseH Reverse Transcriptase (200 U/µg RNA, Invitrogen) and oligo dTs as primers. RT reactions were performed for 50 minutes at 37°C. Real‐time quantitative PCR was performed using MyiQ^®^ detection system (Bio‐Rad) in combination with the iTaq Universal SYBR Green Real‐Time PCR Supermix (Bio‐Rad). The thermal cycling program consisted of initial denaturation in one cycle of 3 minutes at 95°C, followed by 45 cycles of 30 seconds at 95°C, 30 seconds at the individual annealing temperature for each primer and 30 seconds at 72°C. Quantification was performed as described before.^23^ A complete list of primers can be found in Table S1.

RT‐PCR of cardiomyocytes was performed as described before. The amplification of the PCR products was performed under the following conditions: 1 minute at 93°C, 1 minute at 53‐62°C (depending on the primer used) and 3 minutes at 72°C. After amplification, reaction products were separated on a 2% agarose gel, stained with SYBR safe DNA gel stain (Invitrogen) and photographed under ultraviolet illumination. The following primers were used: VEGF: forward: TCC ACC ATG CCA AGT GGT; reverse: TCG GGG TAC TCC TGG AAG AT; VEGF receptor type 1: forward: ACA TTG GTG GTG GCT GAC TCT C; reverse: GGT CCT CTC CTT CGG TTG GTA TC; VEGF receptor type 2: forward: GAA GGG TGA GGA AGG AAG AC; reverse: AGT GCC GAC GAG GAT AAT GAC.

### 2.6 microRNA isolation and small RNA sequencing

Left ventricular samples (4‐5/group) were placed in a 1.5 mL Eppendorf LoBind tube containing glass beads (1.7‐2.1 mm diameter, Carl Roth) and 500 µL of VRX buffer (Viogene Biotek). The Eppendorf tube was firmly attached to a SILAMAT S5 vibrator (Ivoclar Vivadent) to homogenize the tissues. Total RNA was isolated using Viogene miTotal RNA Extraction Miniprep System (Viogene Biotek) according to the manufacturer's protocol. RNA concentration was measured with RNA HS Assay Kit with Qubit 3.0 Fluorometer (Thermo Fisher Scientific). RNA Integrity Number (RIN) was determined using RNA ScreenTape system with 2200 Tapestation (Agilent Technologies).

NEBNext Multiplex Small RNA Library Prep Set for Illumina (New England Biolabs) was used for small RNA library construction according to the manufacturer's protocol. Libraries were quantified and qualified using High Sensitivity DNA1000 ScreenTape system with 2200 Tapestation (Agilent Technologies) and dsDNA HS Assay Kit with Qubit 3.0 Fluorometer (Thermo Fisher Scientific). Libraries were pooled and diluted to 1.8 pM for 2 × 43 bp paired‐end sequencing on the NextSeq 550 Sequencing System (Illumina) at the Xenovea Ltd. using 75‐cycle High Output v2 Kit according to the manufacturer's protocol.

### 2.7 Bioinformatics evaluation of the raw small RNA sequencing data

Illumina FastQ Toolkit (version v2.2.0) was used to perform adapter trimming and filtering of raw reads with thresholds of 30 for average Phred quality score and 10 nt for the read length. Quality control analysis of the filtered reads by FastQC (version v0.11.7) was followed by the alignment of the reads to the rat reference genome (Rnor_6.0 NCBI Rattus norvegicus genome assembly) with the use of Bowtie 2 aligner (version 2.2.1).^24^ FeatureCounts software (version v1.6.2)^25^ and the miRBase (release 22.1) Rattus norvegicus reference annotation^26^ were used to count reads that were aligned to mature miR loci. Fold change estimation and differential expression analysis was performed by the DESeq2 (version 1.10.1)^27^ Bioconductor package which implements the necessary statistical algorithms including dispersion estimation and negative binomial distribution based generalized linear model fitting. Those miRs were further analysed that had a possibly protective expression pattern in the IPC and IPoC‐R groups (opposite changes between I/R vs Control and IPC/IPoC‐R vs I/R comparisons) and that with no observed protective expression changes in the IPoC‐NR group.

### 2.8 Prediction of the microRNA‐target interaction network

Expected expression changes of target genes due to miR mediated post‐transcriptional regulation was predicted by the miRNAtarget™ software (https://mirnatarget.com; Pharmahungary), which was validated in previous studies.^28^, ^29^ A miR‐target interaction network was constructed by miRNAtarget™ based on experimentally validated (miRTarBase version 4.5) and predicted (miRDB version 5.0 with score >80.0 and microRNA.org with mirSVR score <−1.2) miR‐target interaction databases.^30^, ^31^, ^32^ Node strength of each target in the network was calculated by summing the weights (1 or −1) of edges representing the predicted interaction between the target and up‐ or down‐regulated miR nodes. EntOptLayout plugin (version 2.1) for the Cytoscape (version 3.6) software was used to visualize the miR‐target interaction network.^33^

### 2.9 Construction of mitochondria related microRNA‐target subnetwork

Rat specific MitoMiner 4.0^34^ dataset downloaded from the MitoMiner website (http://mitominer.mrc-mbu.cam.ac.uk/release-4.0/impi.do) was used to filter mitochondria related genes from the predicted miR‐target list. The downloaded MitoMiner 4.0 dataset was restricted to genes marked as ‘Known mitochondrial’ or ‘Predicted mitochondrial’ in Integrated Mitochondrial Protein Index (IMPI, http://www.mrc-mbu.cam.ac.uk/impi) Version Q2 2018. Ensembl gene identifier,^35^ Rat Genome Database (RGD) identifier,^36^ NCBI identifier, NCBI symbol and NCBI description^37^ were used to compare our predicted miR‐target list and the IMPI list. Mitochondria related miR‐target subnetwork was constructed from target genes with a Gene Ontology mitochondrial annotation or with an IMPI score above 0.7 and from miRs interacting with these mitochondrial targets.

### 2.10 Isolation and cultivation of cardiomyocytes

Ventricular heart muscle cells were isolated from male rats as described in greater detail previously.^38^ Briefly, hearts were excised under deep ether anaesthesia, transferred rapidly to ice‐cold saline and mounted on the cannula of a Langendorff perfusion system. Hearts were perfused first for 10 minutes in a non‐recirculating manner with a calcium‐free perfusion buffer, then for 20‐25 minutes in a recirculating manner in a buffer supplemented with collagenase and 25 µmol/L calcium. Thereafter, ventricular tissue was minced and incubated for another 5 minutes in recirculating buffer. The remaining cell solution was filtered through a 200‐µm nylon mesh. The filtered material was resuspended in buffer with a stepwise increase in calcium and finally transferred to culture medium (M199 supplemented with carnitine (2 mmol/L), creatine (5 mmol/L) and taurine (5 mmol/L).

### 2.11 Determination of cell contraction

Cell shortening was measured as initially described in greater detail.^39^ Briefly, isolated cardiomyocytes were allowed to contract at room temperature and analysed using a cell‐edge detection system. Cells were stimulated via two AgCl electrodes with biphasic electrical stimuli composed of two equal but opposite rectangular 50‐V stimuli of 0.5 ms duration. Each cell was stimulated for 1 minutes at a frequency of 2.0 Hz. Every 15 seconds contractions were recorded. The mean of these four measurements was used to define the cell shortening of a given cell. Cell lengths were measured via a line camera (data recording at 500 Hz). Data are expressed as cell shortening normalized to diastolic cell length (dL/L (%)).

### 2.12 Immunoblotting

Cells or tissue was lysed in lysis buffer [composition: Tris·Cl (50 mmol/L)), pH 6.7, SDS 2% (wt/vol), mercaptoethanol (2% (wt/vol), sodium orthovanadate (1 mmol/L)]. Proteins were separated by SDS‐page gel electrophoresis and transfer of proteins on nitrocellulose. All membranes were saturated with 2% (wt/vol) BSA and incubated with the first antibody for 2 hours. After the membranes were washed, alkaline phosphatase‐labelled goat anti‐rabbit‐IgG antibodies were added for another 2 hours. Staining was visualized by alkaline phosphatase activity, and a densitometric scan was performed thereafter. The specificity of the antibodies was confirmed on samples after sodium dodecyl sulphate‐gel electrophoresis on the basis of the molecular weight. Primary antibodies directed against other proteins analysed in this study were as follows: SR‐Ca‐ATPase (SERCA2; Santa Cruz Biotechnology, Heidelberg, Germany, C‐20), glyceraldehydes‐3‐phosphate dehydrogenase (GAPDH; EMD Bioscience, CB1001. Secondary antibodies directed against rabbit IgG were also purchased from Sigma‐Aldrich.

### 2.13 Statistics

Data are expressed as indicated in the legends. ANOVA and the Student‐Newman‐Keuls test for post hoc analysis were used to analyse experiments in which more than one group was compared. Data sets were initially analysed by a Shapiro‐Wilk test to analyse whether the data are normally distributed. If this was the case, data were analysed either by paired or unpaired t test depending on the study design. If the two groups had different variances (as analysed by the Levene test), the *P*‐value by a Welch test was calculated. In the case that the data were not normally distributed, a Mann‐Whitney test for unpaired samples was performed. A *P*‐value of ≤.05 is always indicated because this was used as the threshold to reject the null hypothesis. Statistical analysis was performed by SPSS (IBM) version 22.0.

## 3 RESULTS

### 3.1 Effect of IPC and IPoC on infarct size, function and hypertrophy

**Figure 2:**
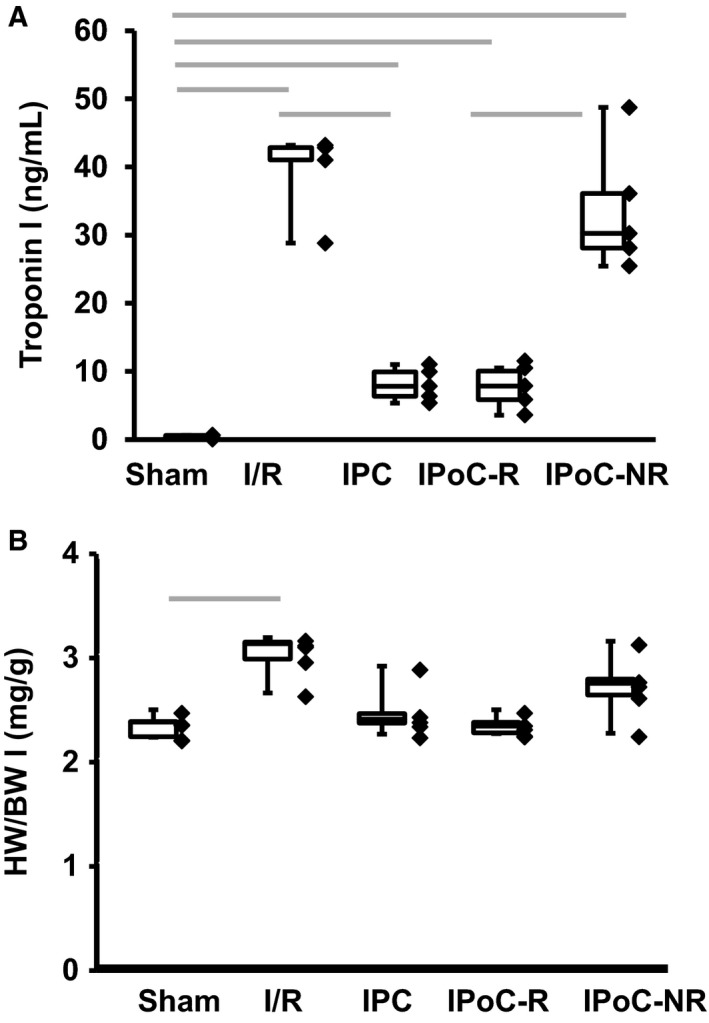
Infarct sizes and hypertrophy in different groups. A, Plasma troponin I levels are shown for all five groups as box and whisker plots with range (whiskers) and 25%, 50% and 75% quartiles. Individual data points are also indicated. B, Heart weight to bodyweight (HW/BW) as box and whisker plots. Grey bars indicate group differences with *P* < .05

In total 80 rats, subdivided in four different treatment groups (Sham, I/R, IPC, IPoC), underwent surgical procedure and plasma troponin I values, as a surrogate parameter for infarct size, was quantified sixty minutes later (Figure 2A). The data revealed the expected increase of plasma troponin I (TnI) after I/R in comparison to sham and reduced plasma levels after IPC in comparison to I/R. However, with regard to IPoC results were heterogeneous. We determined two groups of rats. One had extremely low TnI levels, subsequently termed the IPoC responders (IPoC‐R) and the others had TnI levels indistinguishable from I/R, subsequently termed IPoC non‐responders (IPoC‐NR). During the following week three rats from the sham group and one rat from the IPC group died and could not be considered further. The subsequent analysis was therefore performed with five groups: Sham (n = 17), I/R (n = 20), IPC (n = 19), IPoC‐R (n = 8) and IPoC‐NR (12). Seven days after induction of I/R, the subsequent functional analysis showed increased left ventricular end‐systolic dimension (LVESD) and fractional shortening (FS) after I/R compared to sham (Table 1). In the three protection groups, FS of the IPC group was better than IPoC‐R and both were better than IPoC‐NR, with IPoC‐NR remain indistinguishable from I/R (Table 1). Similarly, I/R and IPoC‐NR had an increased hypertrophy index (heart weight to bodyweight; HW/BW; Figure 2B). However, HW/BW of IPoC‐R but not IPC was indistinguishable from sham. These data suggest that rats of the IPoC‐NR group did not respond to the protocol but also suggests differences between the long‐term adaptation between IPC (better preserved function) and IPoC‐R (less hypertrophy but less effective in terms of function compared to IPC). To analyse the response to post‐infarct remodelling, transcriptional changes in the different tissues were analysed. Therefore, we randomly selected five samples from each group for further analysis. This was done to have equal groups sizes for the subsequent analysis and keeping at least three samples from the smallest group for Western blotting.

**Table 1:** Characterization of left ventricular morphology and function 7 d post‐infarction

	Sham	I/R	IPC	IPoC‐R	IPoC‐NR
HR, bpm	353 ± 44	394 ± 30	421 ± 37	405 ± 28	399 ± 48
AWTd, mm	1.65 ± 0.36	1.75 ± 0.62	2.02 ± 0.23	1.80 ± 0.47	1.87 ± 0.40
AWTs, mm	2.88 ± 0.64	2.78 ± 0.80	3.15 ± 0.69	2.71 ± 0.87	2.99 ± 0.076
PWTd, mm	1.72 ± 0.35	1.78 ± 0.36	1.75 ± 0.37	1.96 ± 0.35	1.90 ± 0.53
PWTs, mm	2.94 ± 0.66	2.91 ± 0.49	2.61 ± 0.57	2.85 ± 0.43	2.85 ± 0.85
LVEDD, mm	4.91 ± 0.73	5.67 ± 1.04	5.01 ± 0.58	5.54 ± 0.63	5.44 ± 0.85
LVESD, mm	1.92 ± 0.77	3.17 ± 1.24**	2.11 ± 0.77	2.67 ± 0.45*	3.23 ± 1.21*
FS, %	61.78 ± 12.49	45.82 ± 13.62**	53.93 ± 16.43	50.24 ± 6.44*	42.11 ± 15.05**
EF, %	92.79 ± 5.18	79.78 ± 14.18**	91.27 ± 6.82	86.05 ± 5.42*	85.76 ± 17.53**

Note

Data are means ± SD.

Abbreviations: AWT, anterior wall thickness; EF, Ejection fraction; FS, fractional shortening; HR, heart rate; LVEDD, left ventricular end‐diastolic dimension; LVSED, left ventricular end‐systolic dimension; PWT, posterior wall thickness.

* *P* < .05 vs Sham.

** *P* < .01 vs Sham.

### 3.2 Effect of IPoC on transcriptome adaptation

Differences in the mRNA expression of different genes between groups are sensitive molecular fingerprints to analyse the effect of protection protocols on the subsequent remodelling.^19^ Our focus was on differences between the IPoC‐R and IPoC‐NR group. As shown in Table 2, among the 10 most strongly I/R‐dependent up‐regulated mRNAs are ones related to extracellular matrix (Collagen‐3, Biglycan, Collagen‐1, Elastin and Fibronectin), inflammation and oxidative stress (matrix metalloprotease (MMP)12, p91^phox^, superoxide (SOD)3) and hypertrophy (atrial natriuretic peptide (ANP), EF‐Hand domain family member D2 (EFhd2). Among the 10 mostly reduced mRNAs are again those coding for proteins linked to modification of the extracellular matrix (Protein phosphatase 1 regulatory subunit 29 (= extracellular leucin‐rich repeat and fibronectin type III domain‐containg protein 2, Elfn2) and Laminin), ion channels (Potassium voltage‐gated channel, subfamily H, member 5, KCNH5; Sodium channel, voltage gated, type VIII, alpha subunit, SCN8a; Sodium‐Myoinositol cotransporter, Slc5a3), signal transduction (Supressor of cytokine signalling 7, Socs7; Forkhead box protein C2, FoxC2), myocardial hypertrophy (Myosin Heavy Chain‐α, Phospholamban) or energy metabolism (PGC‐1α).

**Table 2:** Differentially expressed mRNAs (vs Sham, *x*‐fold, means ± SD) and effect of protection

	I/R	IPC	IPoC‐R	IPoC‐NR	ANOVA
Up					
MMP12	21.18 ± 12.62^a^	1.61 ± 1.51^b^	1.26 ± 0.94^b^	10.68 ± 10.62	*P* = .005
ANP	18.13 ± 1.47^a^	9.57 ± 0.33^a,b^	26.56 ± 15.67^a^	40.00 ± 25.57^a^	*P* = .015
Coll‐3	8.54 ± 1.30^a^	0.96 ± 0.74^b^	0.61 ± 0.25^b^	4.13 ± 3.71	*P* < .000
Biglycan	6.24 ± 1.14^a^	2.92 ± 3.25	1.20 ± 1.10^b^	3.90 ± 2.36	*P* = .004
Coll‐1	5.22 ± 1.40^a^	2.54 ± 2.60	1.14 ± 1.08^b^	2.66 ± 1.73	*P* = .013
Elastin	4.56 ± 0.21^a^	0.82 ± 0.60^b^	0.85 ± 0.52^b^	4.07 ± 1.87	*P* < .000
p91^phox^	4.50 ± 2.20^a^	0.22 ± 0.18	0.09 ± 0.04	0.96 ± 1.33	*P* = .001
EFhd2	3.55 ± 0.93^a^	1.47 ± 0.18	0.48 ± 0.22	1.46 ± 0.84	*P* < .000
SOD‐3	3.22 ± 1.15	3.16 ± 1.87	1.87 ± 1.00	2.94 ± 0.30	*P* = .073
Fibronectin	2.32 ± 0.90	1.49 ± 1.66	0.43 ± 0.46	1.35 ± 0.95	*P* = .132
Down					
Elfn2	0.05 ± 0.01	0.07 ± 0.02	0.03 ± 0.01	0.03 ± 0.01	*P* = .080
Kchn5	0.06 ± 0.03^a^	0.20 ± 0.07^a,b^	0.12 ± 0.04^a^	0.27 ± 0.23^a^	*P* = .013
Scn8a	0.08 ± 0.01	0.21 ± 0.03^b^	0.06 ± 0.01	0.09 ± 0.05	*P* = .041
Socs7	0.13 ± 0.04	0.07 ± 0.04	0.02 ± 0.00	0.05 ± 0.00	*P* = .035
FOXc2	0.16 ± 0.02	0.09 ± 0.07	0.04 ± 0.02	0.08 ± 0.07	*P* = .049
Slc5a3	0.17 ± 0.05^a^	0.26 ± 0.10^a^	0.10 ± 0.02^a^	0.19 ± 0.13^a^	*P* = .002
MHC‐α	0.28 ± 0.08	0.49 ± 0.39	0.76 ± 0.31	0.43 ± 0.21	*P* = .072
PGC‐1α	0.48 ± 0.19	0.42 ± 0.24	0.70 ± 0.14	0.60 ± 0.19	*P* = .094
Laminin	0.49 ± 0.06^a^	0.37 ± 0.15^a^	0.36 ± 0.04^a^	0.49 ± 0.10^a^	*P* = .019
PLB	0.50 ± 0.05	2.05 ± 1.84	2.15 ± 0.58	1.23 ± 0.93	*P* = .222

Abbreviations: ANP, atrial natriuretic peptide; Coll, collagen; Elfn, extracellular leucin‐rich repeat and fibronectin type III domain‐containg protein 2; Fox, forkhead box protein; Kchn, Potassium voltage‐gated channel, subfamily H; MHC, myosin heavy chain; MMP, matrix metalloprotease; PGC, PPAR gamma co‐activator; PLB, phospholamban; Scn, Sodium channel, voltage gated; Slc, Sodium‐Myoinositol cotransporter; Socs, Supressor of cytokine signalling; SOD, superoxide dismutase.

**Figure 3:**
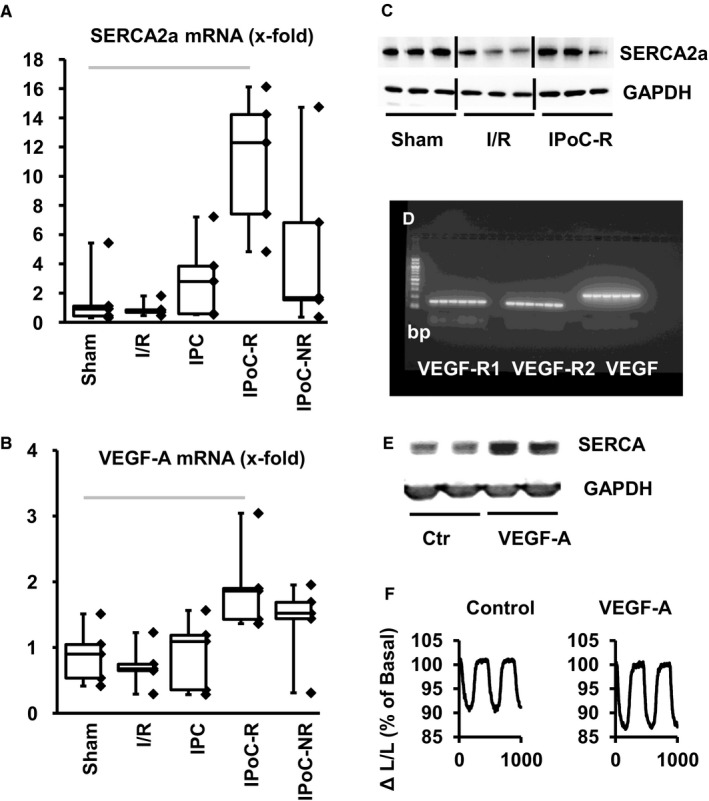
Effect of VEGF‐A on SERCA expression and functional improvement. A + B, mRNA expression of SERCA2a (A) and VEGF‐A (B) in the five groups expressed as box and whisker plots and individual data points. C, Immunoblot of SERCA2a expression and a loading control (GAPDH). D, mRNA expression of VEGF receptor 1 and 2 and VEGF‐A in cardiomyocyte preparations. E, Immunoblot indicating protein levels of SERCA2a 24 h after incubation with VEGF‐A (50 µg/mL); F, Effect of VEGF‐A on load‐free cell shortening expressed as shortening amplitude · 100/ diastolic cell length (ΔL/L)

We next analysed how IPoC and in comparison IPC affected the transcriptional regulation (Table 3). At first, three mRNAs were similarly affected by IPoC‐R, IPoC‐NR and IPC, namely arrestin‐β_2_, forkhead box protein N3 (FoxN3) and Zinc‐finger and BTB‐containing protein 20 (Zbtb20). Three other mRNAs were commonly regulated by IPoC‐R and IPoC‐NR, namely vascular endothelial growth factor‐A (VEGF‐A), EF‐Hand domain family member D2 (EFhd2), and G‐protein receptor coupled kinase‐2 (GRK‐2). These data show that the mRNA expressions of six proteins are similarly affected by IPoC‐R and IPoC‐NR, excluding a complete non‐responsiveness of IPoC‐NR rats. Furthermore, we identified seven mRNAs that are affected by IPoC‐R and IPC. The regulation of these proteins is most likely linked to the regulation of infarct size. Among them, arginase‐1 needs notification as it is linked to the functional post‐ischaemic recovery. Finally, IPoC‐R exclusively regulated 23 genes that were not modified by either IPoC‐NR or IPC, suggesting a specific molecular adaptation of the heart to successful IPoC protection rather than a simple adaptation to smaller infarcts. Most strongly, IPoC increased the expression of mRNA expression of SERCA2a (Figure 3A) and vascular endothelial growth factor (VEGF)‐A (Figure 3B). This finding, the strongest signal in IPoC‐R, was further validated on the protein level (Figure 3C). We verified that adult rat ventricular cardiomyocytes constitutively express VEGF and VEGF receptors (Figure 3D) and found that VEGF induced the expression of SERCA2a (Figure 3E) and increased load‐free cell shortening (Figure 3F) when administered for 24 hours. Load‐free cell shortening was increased by 8.3% from 10.30 ± 0.25% of diastolic cell length to 11.16 ± 0.19%.

**Table 3:** Genes that are differentially expressed by IPoC‐R and IPoC‐NR and its comparison to IPC

Protein	I/R	IPoC‐R	IPoC‐NR	IPC
Regulated by all three conditionings (n = 3)				
Arrestin‐β_2_	1.97 ± 0.42	0.38 ± 0.36***	0.71 ± 0.50*	0.79 ± 0.55*
FOXn3	1.48 ± 0.18	0.68 ± 0.35**	0.74 ± 0.38*	0.55 ± 0.17***
Zbtb20	0.77 ± 0.14	0.35 ± 0.19*	0.37 ± 0.16*	0.45 ± 0.15*
Selectively Regulated by IPoC‐R and IPoC‐NR but not by IPC (n = 3)				
EFhd2	2.49 ± 0.66	0.33 ± 0.15**	1.03 ± 0.59*	1.03 ± 0.83
GRK2	1.39 ± 0.13	0.05 ± 0.02***	0.46 ± 0.31**	0.95 ± 0.51
VEGF‐A	0.69 ± 0.04	1.60 ± 0.23**	1.65 ± 0.20**	0.90 ± 0.50
Selectively Regulated by IPoC‐R and IPC (n = 7)				
Coll‐3	4.26 ± 0.65	0.30 ± 0.13**	2.06 ± 1.85	0.48 ± 0.37**
Elastin	3.02 ± 0.14	0.56 ± 0.34***	2.69 ± 1.24	0.54 ± 0.40**
Psme3	1.31 ± 0.34	0.61 ± 0.27*	0.74 ± 0.24	0.60 ± 0.10*
Arginase‐1	1.18 ± 0.18	0.14 ± 0.05**	1.02 ± 0.77	0.33 ± 0.19**
CaSR	0.89 ± 0.34	0.05 ± 0.01*	0.32 ± 0.22	0.15 ± 0.09*
Bcl‐2	0.82 ± 0.15	0.36 ± 0.09*	0.75 ± 0.05	0.36 ± 0.09*
Intermedin	0.71 ± 0.30	0.03 ± 0.01*	0.35 ± 0.31	0.09 ± 0.05*
Selectively Regulated by IPoC‐R (n = 21)				
Biglycan	4.64 ± 0.85	0.89 ± 0.82***	2.90 ± 1.75	2.17 ± 2.42
Coll‐1	3.62 ± 0.97	0.79 ± 0.75**	1.85 ± 1.20	1.76 ± 1.80
NOX2	2.43 ± 1.19	0.05 ± 0.02*	0.52 ± 0.72	0.12 ± 0.10
Fibronectin	2.13 ± 0.83	0.40 ± 0.42*	1.24 ± 0.87	1.36 ± 1.52
JDP2	1.76 ± 0.47	0.44 ± 0.30**	1.11 ± 0.65	1.52 ± 0.45
TGF‐β_1_	1.71 ± 0.15	0.54 ± 0.32**	1.24 ± 0.67	1.36 ± 0.13
RAMP‐1	1.32 ± 0.39	0.17 ± 0.07*	0.77 ± 0.51	0.29 ± 0.09
PTHrP	1.24 ± 0.52	0.20 ± 0.06*	0.76 ± 0.11	0.62 ± 0.35
RAMP‐3	1.17 ± 0.43	0.29 ± 0.09*	0.74 ± 0.50	1.19 ± 0.73
iNOS	1.09 ± 0.03	0.39 ± 0.14**	0.92 ± 0.29	0.77 ± 0.34
Arginase‐2	1.05 ± 0.18	0.21 ± 0.10***	0.61 ± 0.35	0.80 ± 0.48
Arrestin‐β_1_	1.05 ± 0.35	0.29 ± 0.31*	0.58 ± 0.29	0.49 ± 0.27
eNOS	1.03 ± 0.08	0.50 ± 0.11***	0.73 ± 0.20	0.74 ± 0.48
RAMP‐2	0.98 ± 0.35	0.26 ± 0.05*	0.65 ± 0.32	0.74 ± 0.50
GRK5	0.97 ± 0.15	0.28 ± 0.13***	0.59 ± 0.28	0.76 ± 0.38
MDM2	0.94 ± 0.17	0.48 ± 0.22*	0.67 ± 0.36	1.25 ± 0.64
SDF‐1α	0.90 ± 0.15	0.29 ± 0.21**	0.50 ± 0.20	1.21 ± 1.07
Oxsr1	0.75 ± 0.18	0.36 ± 0.15*	0.48 ± 0.28	0.41 ± 0.19
Ppargc1b	0.71 ± 0.25	0.23 ± 0.15*	0.25 ± 0.16	0.31 ± 0.09
SERCA2a	0.70 ± 0.16	9.74 ± 4.74*	5.02 ± 5.34	2.99 ± 2.47
AR‐B2	0.68 ± 0.04	0.26 ± 0.06***	0.68 ± 0.27	0.68 ± 0.04
PLB	0.41 ± 0.04	1.74 ± 0.47	0.99 ± 0.75	1.66 ± 0.49
Socs7	0.19 ± 0.05	0.05 ± 0.03*	0.12 ± 0.12	0.19 ± 0.18

Abbreviations: AR, adrenoceptor; bcl, B‐cell lymphoma; CaSR, calcium sensing receptor; Coll, collagen; EFhd, EF‐hand domain; FOX, forkhead box protein; GRK, G‐protein coupled receptor; JDP2, jun dimerization protein; MDM mouse double minute; NOS, nitric oxide synthase; NOX, NADPH oxidase; Oxsr, oxidative stress response; PLB, phospholamban; Ppargc, peroxisome proliferator‐activated receptor gamma co‐activator gamma; Psme, proteome activator subunit; PTHrP, parathyroid hormone‐related protein; RAMP, receptor activator modifying protein; SDF, stromal derived factor; SERCA, sarcoplamatic reticulum receptor calcium ATPase; Socs, Supressor of cytokine signalling; TGF, transforming growth factor; VEGF, vascular endothelial growth factor; Zbtb, Zinc‐finger and BTB‐containing protein.

* *P* < .05 vs I/R

** *P* < .01 vs I/R

*** *P* < .001 vs I/R.

Furthermore, we identified JDP2, an inhibitor of activator protein‐1 (AP‐1), to be specifically down‐regulated in IPoC‐R. JDP2 showed the strongest difference between IPoC‐R and IPoC‐NR. This suggests that down‐regulation of JDP2 and thereby activation of AP‐1 may be involved in the process of adaptation in IPoC‐R.

### 3.3 Effect of IPoC on mitochondrial proteins

**Figure 4:**
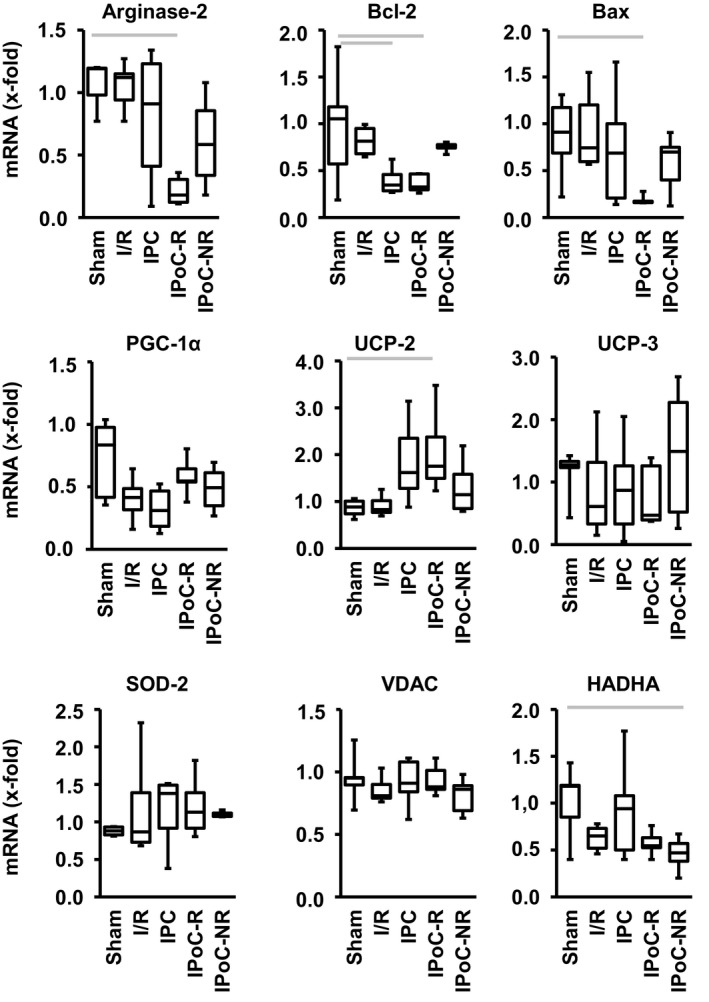
Differential mRNA expression of mitochondrial proteins in post‐infarct hearts. Data are given as box and whisker plots. Grey bars indicate group differences with *P* < .05

Next, we looked on mitochondrial proteins in more depth as mitochondria are key regulators of cardiac protection.^40^ IPoC‐R differed from other groups in three aspects: Only IPoC‐Rs down‐regulated the mRNA expression of arginase‐2 and bax and increased the expression of uncoupling protein (UCP)‐2 (Figure 4). The latter one was similarly found in IPCs. Group differences did not occur in other aspects such as peroxisome proliferator‐activated receptor gamma co‐activator (PGC)‐1α, UCP‐3, superoxide dismutase (SOD)‐2 and voltage‐dependent anion channel (VDAC). Trifunctional enzyme subunit α (HADHA) showed the strongest down‐regulation in IPoC‐NR (Figure 4).

### 3.4 Effect of IPoC on microRNA transcriptome

**Figure 5:**
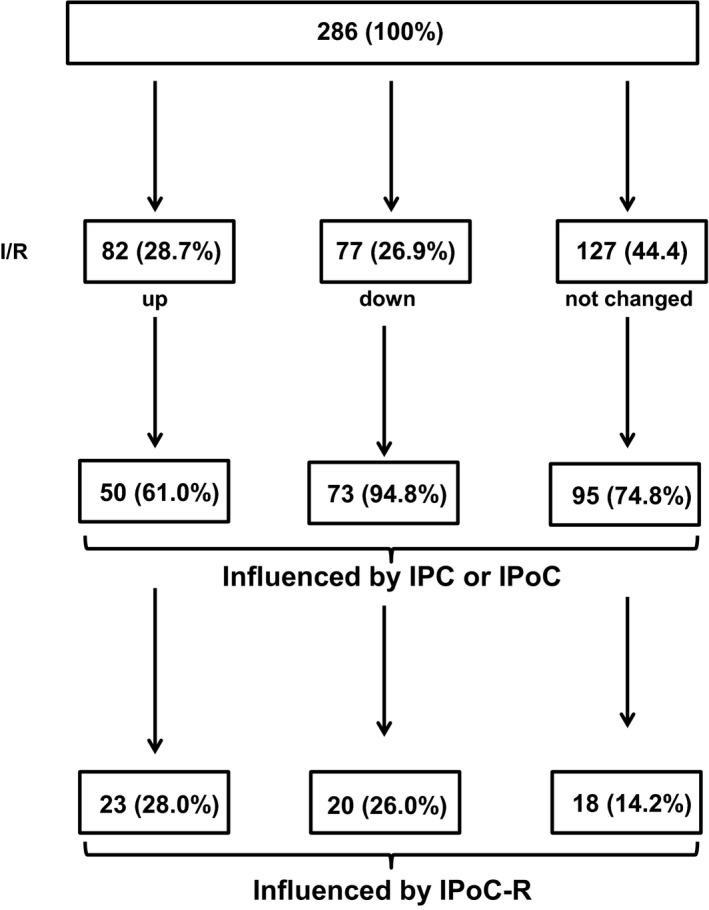
Overview of miRs analysis and comparison of overall changes on the basis of differences producing a *P* < .05

The regulation of mRNA expression depends on the activation of transcription factors that modify the de novo synthesis of mRNA. Furthermore, the mRNA expression also depends on mRNA stability that can be targeted by miRs. We, therefore, extended our analysis and investigated the effect of IPoC on miRs. In total, 627 miRs were read and 286 miRs showed differential expression if only *P*‐values were considered, from which 28.7% (n = 82) were up‐regulated by I/R (Figure 5). Among them were miR‐34b‐3p, miR‐34c‐3p, miR‐195‐3p, miR‐130b‐5p and miR‐155‐5p that were previously also found in pigs therefore are evolutionary conserved. From these miRs 28% (n = 23) were differentially regulated in the IPoC‐R group. The strongest effects were on miR‐199a‐3p and miR‐148a‐5p (*P* < .01). Of note, both were not regulated by IPC. Only one of IPoC‐dependent down‐regulated miRs that are normally increased by I/R was down‐regulated in all three conditioning groups (miR‐122‐5p). In only one case, the down‐regulation by IPoC‐R was found for IPC but not IPoC‐NR (rno‐miR‐34c‐5p). In four cases, the expression was even further enhanced in IPoC‐NRs (miR‐455‐5p, miR‐195‐3p, miR‐271a‐5p and miR‐148a‐3p).

I/R caused also a down‐regulation of 26.9% (n = 77) miRs. Nearly all of them were affected by any conditioning method (94.8%). This effect was influenced in IPoC‐R in 26.0% (n = 20). In six cases, this was also seen in IPC‐treated rats, therefore linked to smaller infarcts rather than to specific effects of IPoC. In one case, down‐regulation was enhanced in IPoC‐NRs (miR‐133a‐3p). At last, 18 miRs that were not differentially expressed by I/R but either reduced (n = 10) or increased (n = 8) in the IPoC‐R. The strongest down‐regulated miR induced one was miR‐532‐5p. Neither IPC nor IPoC‐NR influenced the expression of these miRs.

### 3.5 Effect of IPoC on mitochondrial related microRNA‐target subnetwork

**Figure 6:**
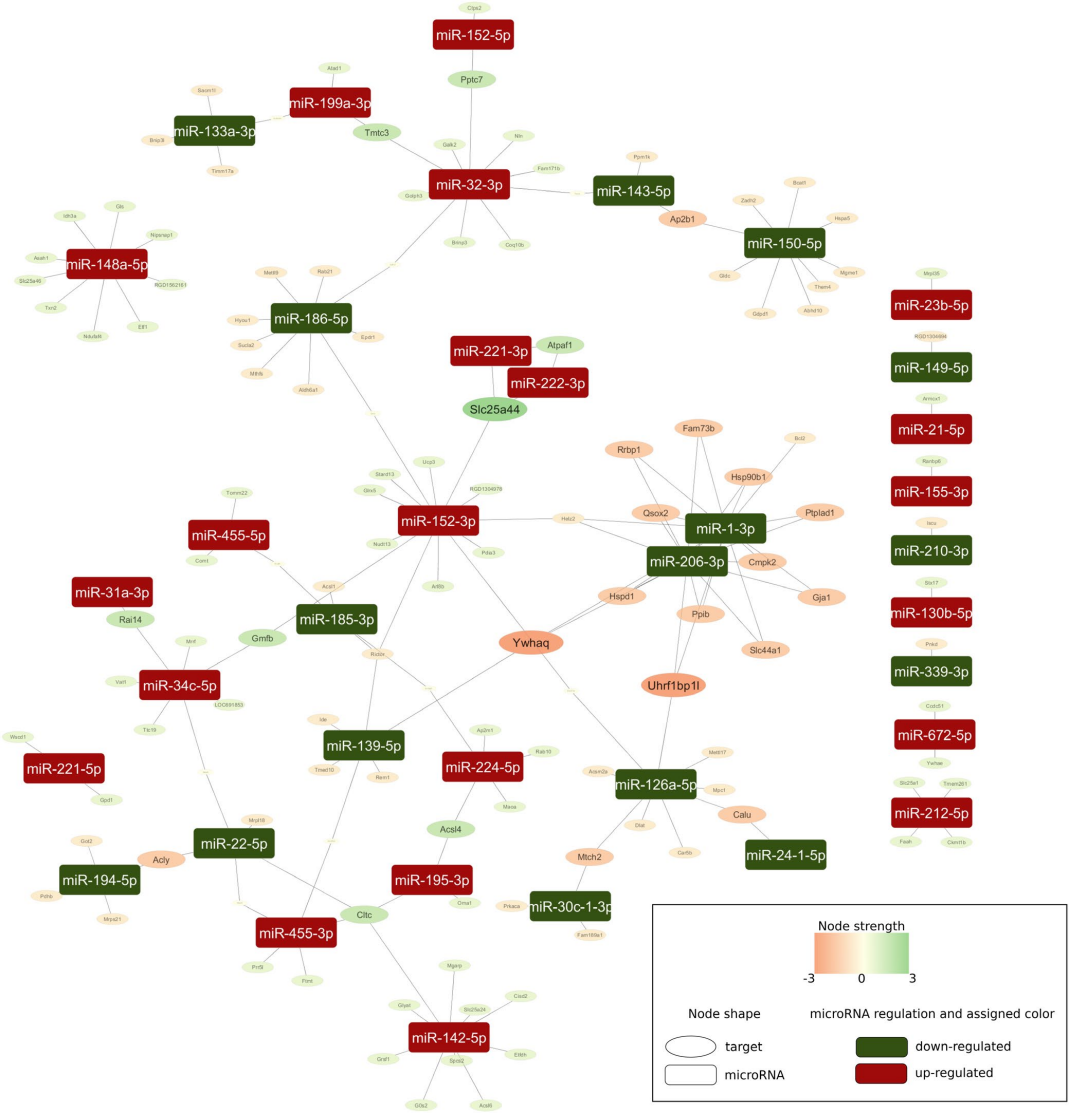
Mitochondrial‐associated miRNAs and linkage to potential targets found in post‐ischaemic hearts

As mentioned before, mitochondrial proteins are significantly affected by IPoC. To understand the contribution between miR regulation and mitochondrial transcriptome in more depth, mitochondrial related miR‐target subnetworks were analysed. In total, we found 21 miRs that are up‐regulated by I/R and affected by IPoC. Subsequent network analysis identified UHRF1 binding protein 1‐like (Uhrf1bp1l), tyrosine 3‐monooxygenase/tryptophan 5‐monoxygenase activation protein theta (Ywhaq) and solute carrier family 25, member 44 as those targets with the highest strength (Figure 6).

## 4 DISCUSSION

The most important finding of our study is that IPoC in healthy rats leads to a heterogeneous responsiveness as monitored by an infarct size surrogate parameter. Even in those cases in which IPoC was associated with small infarcts, IPoC differed from IPC, which was always cardioprotective in the same animal model. The main difference was less hypertrophy (as measured by heart weight to bodyweight). However, similarities between IPoC‐R and IPoC‐NR in the subsequent remodelling process as seen by equal regulation of some mRNAs indicate that the responsiveness to IPoC in IPoC‐NRs is rather incomplete than completely missing. Previous studies reported about differences in the anti‐arrhythmic property of IPoC and IPC.^41^ Thus, differences between both types of cardioprotection in long‐term outcome have been reported before as well. IPoC activates so called salvage pathways that are also involved in cardiac remodelling.^42^ IPoC activates pathways that are involved in the cell damage as monitored by infarct size as well as in subsequent remodelling that depends only in part on the infarct size. We suggest that those parts of the effects of IPoC on subsequent remodelling are still active in hearts without effective cardioprotection quantified by infarct size. This holds for VEGF/SERCA2a pathways. Similarly, in pig hearts IPoC could protect the microvasculature without lowering the infarct size.^43^ However, our data show that without effective infarct size lowering, IPoC is insufficient to promote adaptive remodelling and preserve function. As a main finding of this study, mitochondrial proteins showed a divergent regulation on the melev of miR and mRNA between IPoC‐R and IPoC‐NR.

In this study, we grouped IPoC‐treated rats into responders and non‐responders based on TnI plasma levels. In terms of cardioprotection by conditioning, this is not the gold standard. However, we previously showed that plasma TnI correlates with infarct size as quantified by TTC staining that served as gold standard.^20^, ^44^ Furthermore, IPC successfully protected the hearts but a heterogeneous distribution was found only for IPoC suggesting that inter‐individual responsiveness of rats is not a technical problem but related to different biological responsiveness. In this study we extended the post‐infarct analysis to a time‐period beyond the early minutes of reperfusion.

Different results concerning the ability of IPoC to protect rats are well known from recent publications. They report no protection or complete effectiveness (see Introduction). Differences in animal strains, animal handling and other variables may account for this. Our study standardized these variables as best as possible. However, although in principle the IPoC protocol was sufficient to protect rats, IPoC reduced infarct size not in all rats, indicating an endogenous heterogeneity. Our study does not identify a master molecule that is responsible for this difference. However, strong differences were found between IPoC‐R and IPoC‐NR for the expression of JDP2 mRNA. JDP2 is an intrinsic inhibitor of the activator protein‐1 (AP‐1). Transgenic over‐expression of JDP2 reduces the expression of SERCA2a thereby impairing the responsiveness to β‐adrenoceptor stimulation.^45^ In the current study we found a down‐regulation of JDP2 in IPoC‐R and *vice versa* a strong up‐regulation of SERCA2a (mRNA and protein). Another mRNA similarly strongly regulated by IPoC was VEGF. At least in endothelial cells, VEGF induction is AP‐1‐dependent.^46^ Here we show that adult rat ventricular cardiomyocytes express VEGF receptors and VEGF, that VEGF can induce the protein expression of SERCA2a in cultured adult rat ventricular myocytes and increase cell function. In IPoC‐NR SERCA2a and VEGF‐A expression were less activated. It might be that basal expression of JDP2 is responsible for the different responsiveness of rats to IPoC. Experiments with transgenic mice over‐expressing JDP2 will clarify this question in the future. If JDP2 is a key molecule for the success of IPoC than its increased expression should attenuate cardioprotection by IPoC in mice as well. Further support to this hypothesis comes from previous findings that AP‐1‐dependent regulated genes such as arginase‐1 are indeed the first that are differentially regulated in a post‐infarct heart and responsible for the functional recovery of post‐infarct hearts via expression of arginase‐1.^22^

In the current study, we found a strong induction of VEGF‐A and SERCA2a expression. Subsequent analysis showed that myocytes respond to VEGF by improved expression of SERCA2a and improved cell function. However, despite this protective effect, overall function in IPoC‐treated rats, and more specifically in IPoC‐NRs, indicates that these cellular improvements are not sufficient in the whole heart to stabilize post‐infarct function. As proper heart function depends on energy supply, we subsequently analysed the mRNA expression of mitochondrial proteins in more deep. Interestingly, we found major differences between IPoC‐R and IPoC‐NR with respect to the expression of arginase‐2, bax and UCP‐2. Arginase‐2, the mitochondrial‐specific isoform of arginase, is known to trigger maldaptive effects due to ischaemia in the retina and kidney. The main function of this enzyme is to affect polyamine metabolism. In this context, down‐regulation of arginase‐2 as seen in IPoC‐Rs reduces polyamine metabolism required for cell growth. In this context, it might be relevant that IPoC‐R hearts are smaller than I/R hearts. There are currently no data available concerning the role of arginase‐2 in ischaemic conditioning. Our data suggest that down‐regulation of arginase‐2 is specific for IPoC but only successful in IPoC‐Rs. Bax is a key protein controlling the cellular apoptosis by regulating cytochrome C release from mitochondria. In the current study, we show a specific down‐regulation of bax in IPoC‐Rs. The role of UCP‐2 in mitochondria is not completely clear. As a homologue of UCP‐1 the structure and name of the molecule suggest a role as uncoupling protein, that is that electron transport chain of mitochondria is bypassed. As a result, it is expected that UCPs reduce oxidative stress in mitochondria but decrease ATP production.^40^ However, alternative functions of UCP‐2 have been proposed due to its low uncoupling efficiency.^47^ Among them, UCP‐2 may attenuate glucose uptake. Interestingly, UCP‐2 was up‐regulated in post‐infarct hearts only in IPC and (more significant) in IPoC‐R. Both groups had the best functional recovery suggesting that at least in the post‐infarct hearts UCP‐2 expression is required for improvement of function. One may speculate that increasing the expression of UCP‐2 in these hearts stabilizes metabolic flexibility in rat hearts.

Changes in the mRNA expression of molecules that participate in processes such as fibrosis, inflammation and electrophysiology are well described in post‐infarct hearts and therefore it is not surprising that we found multiple changes in these rats after seven days. However, the underlying level of regulation is less known. In principle, this can be performed by activation of transcription factors or modulation of miRs that target specific mRNAs, thereby shorten their halflife and expression level. IPC and IPoC affect the expression of miRs within 2 hours of reperfusion but whether this is relevant for the subsequent remodelling process remained unclear.^48^ In response to I/R we found 82 miRs up‐regulated among them were five previously also found in a pig model.^43^ This indicates a strong evolutionary regulation and a common cluster of miRs and subsequent mRNAs affected by I/R. However, in the current study changes between I/R and IPoC‐R were in the focus. Here 199a‐3p and miR‐148a‐5p showed the greatest effects. Both miRs were induced by I/R and this induction was attenuated by IPoC‐R. Transgenic mice with disruption of miR‐199 showed an adaptive type of hypertrophy and increased expression of PGC‐1α.^49^ Here, we found a down‐regulation of miR‐199 in the IPoC‐R group compared to I/R and an increase in PGC‐1α (from 0.48 ± 0.19 to 0.70 ± 0.14). Furthermore, a down‐regulation of miR‐199 was linked to an increased activity of the proteasome systems in end‐stage dilative cardiomyopathy.^50^ In our study, IPoC‐R group were unable to increase their heart weights but had the largest left ventricular end‐diastolic dimensions among the protection groups. The lack of hypertrophic responsiveness to IPoC in responders may be the consequence of increased activity of the proteasome linked to miR‐199 down‐regulation as well. Finally, miR‐199 was increased in plasma samples from patients with acute myocardial infarction and experiments with H9c2 cells further suggesting a protective role for miR‐199 down‐regulation against hypoxia‐dependent cytotoxicity.^51^ In hepatocellular carcinoma cells, miR‐199a‐3p suppresses VEGF release and VEGF receptor expression ^52^. This suggests that miR‐199 down‐regulation in the IPoC‐R participates in the subsequent protective remodelling including VEGF‐dependent pathways. These suggestions make miR‐199 an interesting candidate to be targeted by IPoC or alternatively by other procedures. MiR‐148a‐5p, however, has not yet been associated with cardiovascular diseases but may require more attention in future studies.

Detailed analysis of mitochondrial network pathways identified solute carrier family 25, member 44 (Slc25a44) as the main target mRNA that by prediction should be down‐regulated. Interestingly, Slc25a44 is linked to miR‐222‐3p. This miR is induced by I/R and in IPoC‐NRs but not in IPC and IPoC‐R. Collectively, these data suggest an important role for miRs for the regulation of mitochondrial‐specific genes in I/R.

In conclusion, this study shows that IPoC‐NR (as classified by infarct size) still react to IPoC by molecular adaptations distinct from IPC and I/R but comparable to IPoC‐R. This adaptation, however, is incomplete. We found inter‐individual variability in response to IPoC in healthy rats specifically in mitochondrial‐specific adaptations suggesting that non‐responsiveness is not simply linked to co‐morbidities. A high inter‐individual variability may limit the availability of IPoC in other species as well. However, as certain types of co‐morbidities affect mitochondrial function this may be a link to reduced responsiveness.

## CONFLICT OF INTEREST

The authors declare that they have no conflict of interest.

## AUTHOR CONTRIBUTIONS

RS, JK, HSK, PB, KG, BA and ES performed the experiments. KDS, RS and PF designed the experiments and wrote the paper. RS, KDS, P.B, BB and BA analysed the data. All authors approved the final version of the paper.

## Supporting information

Table S1

## Data Availability

All data needed to evaluate the conclusions in the paper are present in the paper and/or supplements. Additional data related to this paper may be requested by the author.
