# Supplementary material for: Ischaemic post‐conditioning in rats: Responder and non‐responder differ in transcriptome of mitochondrial proteins
Source: J Cell Mol Med. 2020 Apr 16;24(10):5528–41. doi: 10.1111/jcmm.15209 (PMC7214154; doi:10.1111/jcmm.15209)
Supplement: Supplementary file 1 — Table S1 [file JCMM-24-5528-s001.docx]

Supplement Table 1: List of primers used in this study

Forward Reverse

Adrenoreceptor-β_2_ GCT TCT GTG CCT TCG CCG GT AGC CTT CCA TGC CAG GGG CT

ANP ATG GGC TCC TTC TCC ATC AC TCT TCG GTA CCG GAA GCT G

Arginase-1 GGA AGC ATC TCT GGC CAC GCC CAC CGG TTG CCC GTG CAG AT

Arginase-2 TGA GGA GCA GCG TCT CCC GT GCT TCT CGG ATG GCG GCT G

Arrestin-β_1_ GGG CGA CAA AGG GAC ACG A GTG TCA CGT AGA CTC GCC TT

Arrestin-β_2_ TCG AGC CTT CTG TGC CAA AT AGA CAT GAG GAA GTG GCG TG

Bax ACT AAA GTG CCC GAG CTG ATC CAC TGT CTG CCA TGT GGG G

Bcl-2 ATC TTC TCC TTC CAG CCT GA TCA GTC ATC CAC AGA GCG AT

Biglycan TGA TTG AGA ATG GGA GCC TGA G CCT TGG TGA TGT TGT TGG AGT G

CaSR AAG TGC CCG GAT GAC TTC TG GGT TGG TGG CCT TGA CGA TA

Collagen-1 GCG AAC AAG GTG ACA GAG CCA GGA GAA CCA GCA GAG

Collagen-3 TGG AGT CGG AGG AAT G GCC AGA TGG ACC AAT AG

EFhd2 AAC TTC TTC GAG GCC AAG GT TTA AAG GCC GCT TTT CGC TG

Elastin TGC TAC TGC TTG GTG GAG AAT G CGT GGC TGC TGC TGT CTG

Elfn2 TAT GAG ACC ATC CCC CAG CA GTG AGG TTG AGG TCC GTG AG

eNOS CAA CTG GAA AAA GGC AGC CC AAG AGC CTC TAG CTC CTG CT

Fibronectin TGG AGC AAG AAG GAC AAC CGG ACA TCT GTG AAG GAG

FOXc2 GAA GGA CGT GCC CAA GGA TA CTC GCT CTT GAC CAC CAC TTT

FOXn3 GTG GTG CAT AGA CCC AGA GT CTG GAG AAA CTT GCA GAA GGG

GRK2 GCT CTT CAA GTT GTT GCG GG AAA CCT TCC AGC AGG GAT CG

GRK5 GCA ACA TGC TGC TCA CCA AA CGA AGG GAG GGT CCA ACA TC

HADHA ACA TCG GAG CAG TCT TTG GG AGC TGA CAC GGG GTA AAC TG

HPRT CCA GCG TCG TGA TTA GTG AT CAA GTC TTT CAG TCC TGT CC

iNOS AAG AGA CGC ACA GGC AGA G CAG CAG GCA CAC GCA ATG

Intermedin TGC CTC AGG GTG GTG GCT CA ACT GTG GGG GCT GCT GGG AT

JDP2 TCA GAG AGG CTG GAG CTC AT ACT TCT TGT CCA GCT GCT CC

Kchn5 AGA CAT CGC TGG CCT ATG TG GAT GGG GCT GTT GAC CTT GA

Laminin CGA GGA TGT CAG CGT TGT C TCA CAG CCG TCT CCA GTC

MDM2 TGT CTG TGT CTA CCG AGG GT GGG ACT CCG AAC ACA TCT CC

MHC-α CAC CCT GGA GGA CCA GAT TA TGG ATC CTG ATG AAC TTC CC

MMP12 TGC AGC TGT CTT TGA TCC AC GCA TCA ATT TTT GGC CTG AT

Oxsr1 CTG GTC GAC GGA AGG GAT TT CCG AGC CTT CAA CAC CAG AT

p91^phox^ GTT TGC CGG AAA CCC TCC TA CCT TCT GCT GAG ATC GCC AA

PGC-1α AGT GCT CAG CCG AGG ACA CGA TGC CCC TGC CAG TCA CAG GA

Phospholamban TAT GTC TGC TGC TGA TAT GC ACT CTT AAA TCG TGA CCC TTC

Ppargc1b GGC CAC ACC TGT CTA TGC TT AGG CTT GTT GAC ATC CCG TT

Psme3 GAT TCG GCT GCT GAT CGA GA TGG TCC AGA TAA GAC GCA GC

PTHrP AGC TAC TCC GTG CCC TCC CG AGG AAG AAA CGG CGG CGC AA

RAMP-1 AGC ATC CTC TGC CCT TTC ATT GAC CAC CAG GGC AGT CAT G

RAMP-2 GCA GCC TAC CTT CTC CGA TCC TCC TCC ACA CCA CAA GCG TAA C

RAMP-3 CAA CCT GTC GGA GTT CAT CGT TGT CTC CAT CTC CGT GCA GTT

Scn8a CCA GAA GAA CGG GAA CGG AA ACG GTC AGG TTT GGG TTG TT

SDF-1α CCA AGG TCG TCG CCG TGC TG GGC TCT GGC GAC ATG GCT CT

SERCA2a CGA GTT GAA CCT TCC CAC AA AGG AGA TGA GGT AGC GGA TGA A

Slc5a3 GCA TTA AGG GGC TCC AAC CT ATG AGA GCT GCT TGC CCA TT

Socs7 AGG AGG AGG ACG CGG AG CTC TAG TCC AGC CAC CGG A

SOD-2 ATG TTG TGT CGG GCG GCG TG TCG CGT GGT GCT TGC TGT GG

SOD-3 TGC TGC CTC CCG ATC AGC CA CCC TGG CTC AGG TCC CCG AA

TGF-β_1_ ATT CCT GGC GTT ACC TTG G CCT GTA TTC CGT CTC CTT GG

UCP-2 CAC CGT CAT TGC CTC CCC CG CGG AGC ATG GTC AGG GCA CA

UCP-3 GAT CTC CTC ACC TTC CCC CT AGG CAA AAC TCA TCT GGC GA

VEGF-A TGC CCC TAA TGC GGT GTG CG GGC TCA CAG TGA ACG CTC CAG G

VDAC CTA CGG GCT CAT CTT CAC CC TGT CAA CGT TAC TGC CCA CA

Zbtb20 ACC AGC TAG AAA CAG GTG CC TGT AGG ACG CCC TTA TCG GA
